# Update on the Molecular Aspects and Methods Underlying the Complex Architecture of FSHD

**DOI:** 10.3390/cells11172687

**Published:** 2022-08-29

**Authors:** Valerio Caputo, Domenica Megalizzi, Carlo Fabrizio, Andrea Termine, Luca Colantoni, Carlo Caltagirone, Emiliano Giardina, Raffaella Cascella, Claudia Strafella

**Affiliations:** 1 Genomic Medicine Laboratory-UILDM, Santa Lucia Foundation IRCCS, 00179 Rome, Italy; 2 Department of Biomedicine and Prevention, Tor Vergata University, 00133 Rome, Italy; 3 Data Science Unit, Santa Lucia Foundation IRCCS, 00179 Rome, Italy; 4 Department of Clinical and Behavorial Neurology, Santa Lucia Foundation IRCCS, 00179 Rome, Italy

**Keywords:** FSHD, *DUX4*, muscular distrophy, genomics, transcriptomics, (epi)genetics, nc-RNA, single-cell RNA-seq, NGS, artificial intelligence, machine learning

## Abstract

Despite the knowledge of the main mechanisms involved in facioscapulohumeral muscular dystrophy (FSHD), the high heterogeneity and variable penetrance of the disease complicate the diagnosis, characterization and genotype–phenotype correlation of patients and families, raising the need for further research and data. Thus, the present review provides an update of the main molecular aspects underlying the complex architecture of FSHD, including the genetic factors (related to *D4Z4* repeated units and FSHD-associated genes), epigenetic elements (*D4Z4* methylation status, non-coding RNAs and high-order chromatin interactions) and gene expression profiles (FSHD transcriptome signatures both at bulk tissue and single-cell level). In addition, the review will also describe the methods currently available for investigating the above-mentioned features and how the resulting data may be combined with artificial-intelligence-based pipelines, with the purpose of developing a multifunctional tool tailored to enhancing the knowledge of disease pathophysiology and progression and fostering the research for novel treatment strategies, as well as clinically useful biomarkers. In conclusion, the present review highlights how FSHD should be regarded as a disease characterized by a molecular spectrum of genetic and epigenetic factors, whose alteration plays a differential role in *DUX4* repression and, subsequently, contributes to determining the FSHD phenotype.

## 1. Introduction

**Figure 1:**
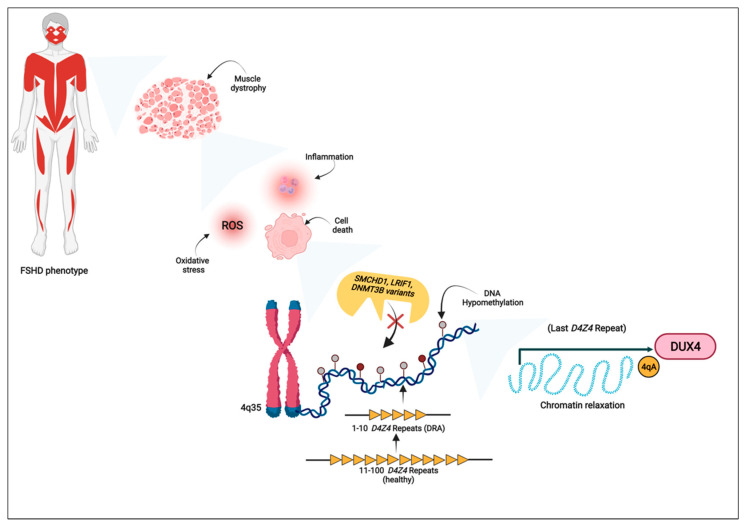
The main disease mechanisms and molecular determinants involved in FSHD etiopathogenesis. The figure illustrates how the FSHD phenotype results from the muscle dystrophy and dysfunction, which, in turn, are due to altered biological mechanisms such as cell death, inflammation and oxidative stress. The dysregulation of such pathways has been associated with DUX4 toxic expression. In presence of a 4qA permissive allele, *DUX4* activation depends on the chromatin relaxation of the *D4Z4* array that can result from the partial deletion of the *D4Z4* repeated units, the occurrence of pathogenic variants within *SMCHD1*, *LRIF1* and *DNMT3B* genes and the concomitant DNA hypomethylation. DRA: *D4Z4* reduced allele; ROS: reactive oxygen species. Created with Biorender.com, accessed on 15 July 2022.

Facioscapulohumeral muscular dystrophy (FSHD) represents the third most common dystrophy, affecting 1:8333 individuals worldwide. The disease is generally characterized by a progressive weakness involving the muscles of the face and the upper and lower extremities [1,2,3], although a wide range of mild-to-severe phenotypes are observed [4]. Moreover, extra-muscular phenotypes are known, such as hearing loss and retinal vascular pathologies. The onset of FSHD is usually between the second and third decade of life, although infantile or late-onset cases can also occur [5]. The main mechanisms underlying FSHD (Figure 1) refer to the epigenetic derepression of the Repeated Units (RU) that form the *D4Z4* macroarray (4q35), which, in turn, are responsible for the aberrant expression of DUX4.

Over the *D4Z4*, similar macroarrays have been reported in other chromosomes. In particular, the 10q26 region harbors an array that shares 98% of the *D4Z4* sequence of the 4q35, though the latter is the only one associated with the disease pathogenesis [6]. Notably, the *DUX4* gene is located within each RU of the *D4Z4* and encodes a transcription factor involved in the genome activation of zygotes at the very early stage of organism development. However, its expression in adult differentiated muscle cells was found to cause the dysregulation of gene expression, leading to apoptosis, the induction of oxidative stress and inflammatory pathways [2,3,5]. Intriguingly, DUX4 was reported to induce apoptosis by a p53-dependent mechanism in murine and zebrafish models [7], although other studies found that the p53 activity was not relevant in both mice and cells induced to express DUX4 [8,9].

Considering that the disease is mainly related to the toxic expression of *DUX4*, the epigenetic machinery (including DNA methylation, histone post-translational modifications, chromatin conformation and post-transcriptional regulators) has also been investigated as a contributing factor to the disease development. Despite this knowledge, the disease mechanisms are still not fully understood. In fact, the high heterogeneity of symptoms and the variable penetrance of the known alterations further complicate the diagnosis, as well as the genotype–phenotype correlation. To date, the molecular diagnosis is essentially based on (i) the *D4Z4* sizing by means of linear and/or pulsed-field gel electrophoresis (LGE/PFGE) and Southern blotting followed by hybridization with specific probes, and (ii) the research for pathogenic mutations within known associated genes by direct resequencing or NGS [5,10].

However, the molecular diagnosis still needs to be improved in terms of precision, accuracy and required times and costs. Indeed, the *D4Z4* sizing is labor-intensive and the targeted sequencing can limit the power of diagnosis. Therefore, novel biomarkers and methodologies that can be applied to the clinical practice are needed to enable a cost-effective and more accurate profiling of FSHD phenotype. On this subject, the identification of disease-specific transcriptome signatures could provide useful markers of disease that are able to improve the characterization and, eventually, support the diagnosis and treatment. Indeed, genetic and molecular analyses performed on easy-to-access samples (such as blood and saliva) could provide valuable information that can be used for clinical purposes, decreasing the cost of tests while maintaining a high informative power. In this regard, advanced computational methods of analysis could be used to better evaluate genetic, epigenetic and transcriptomic data as potential biomarkers for diagnosis and prognosis. For instance, it has been shown how machine learning (ML) approaches can predict a patient’s disease status from molecular data, providing clinicians with computational assistance based on artificial intelligence (AI) [11]. For this purpose, the present review will discuss the molecular aspects (genetic factors, epigenetic elements and gene expression profiles) and methods that can be exploited for clinical purposes and that may be combined with AI-based pipelines to provide a better characterization of FSHD by means of multifunctional tools.

## 2. Genetic Aspects of FSHD

Two genetic forms of FSHD have been traditionally described, namely FSHD1 and FSHD2. Both of them are characterized by an autosomal dominant transmission and overlapping clinical features. The first form is caused by the partial loss of RU within the *D4Z4*, at least in one 4q35 chromosome [6,9,10]. As a matter of fact, healthy individuals have been reported to harbor a number of Rus, ranging from 11 to 100, whereas subjects affected by FSHD1 display 1 to 10 RUs at the *D4Z4* locus (Figure 1). In particular, the reduced number of RUs is generally accompanied by the loss of repressive epigenetic features, which result in the relaxation of chromatin conformation, DNA hypomethylation and histone modifications. Altogether, such alterations are indicative of a transcriptionally active genomic region [3,12,13,14]. The *D4Z4* contraction, together with the presence of the permissive 4qA allele (within a 10 kb sequence in the distal part of subtelomere), leads to the stabilization and expression of the full length *DUX4* transcript (*DUX4*-FL) [6,15]. Notably, the last copy of *DUX4* is localized close to the subtelomeric region and, unlike the copies located within each of the *D4Z4* RUs, the third exon of the last copy harbors a polyadenilation signal (PAS) in the presence of 4qA, which is fundamental for the stabilization of the *DUX4*-related mRNA and its subsequent maturation and translation. Beside the presence of PAS, the 4qA haplotype displays simple sequence length polymorphisms (SSLPs) in the proximity of the *D4Z4* repeat that allow for distinguishing different 4qA subtypes. Of these, the SSLPs 4A161, 4A159 and 4A168 have been found in FSHD1 patients [6]. The contraction of the *D4Z4* array to 1–10 RU, together with permissive 4qA haplotypes, has been reported to account for approximately 95% of all FSHD patients [16].

Given these premises, the “first level” of FSHD diagnosis is currently represented by the detection of a 1–10 RUs contraction at the *D4Z4* locus (referred to as *D4Z4* reduced allele, DRA) and a permissive 4qA subtelomeric haplotype [12,15,17,18]. For this purpose, most laboratories proceed with the digestion of the genomic DNA by the EcoRI enzyme, PFGE, Southern-blot-based analyses and hybridization with a P13-E11 probe. However, such strategies have some limitations, which are mainly due to the fact that Southern blotting is a semi-quantitative method. Therefore, molecular combing (MC) and single-molecule optical mapping (SMOM) have been introduced as alternative methods for FSHD diagnosis. In particular, the MC technique allows for mapping genetic elements such as the *D4Z4* sequence by the direct visualization of multiple DNA molecules at an estimated resolution of 1 kb [19,20,21]. This approach has been reported to be more sensitive and precise than Southern blotting. In particular, it has been able to correctly analyze samples with undetermined results when using the traditional method and to detect rearrangements in a cohort of 87 FSHD subjects [22]. Concerning SMOM, it allows for mapping single DNA molecules by means of fluorescence imaging [23]. This approach has been shown to provide a more precise quantification of the RU number and to detect the presence of mosaicism with respect to the traditional method [24]. Altogether, MC and SMOM represent feasible techniques that are able to improve the precision of *D4Z4* sizing, although further validation on larger cohorts and on patients with complex rearrangements and mosaicism are required.

Over *D4Z4* contraction, FSHD has also been associated with the occurrence of detrimental variants within the sequences of *Structural Maintenance of Chromosomes flexible Hinge Domain–containing protein 1* (*SMCHD1*, 18p11.32) and *DNA Methyltransferase 3 Beta* (*DNMT3B*, 20q11.21) *Ligand Dependent Nuclear Receptor Interacting Factor 1* (*LRIF1*, 1p13.3) genes. The activity of these genes is crucial for maintaining the epigenetic repression of the locus in the presence of 4qA permissive subtelomeres [25,26,27,28,29]. Most of the pathogenic variants associated with FSHD occur within *SMCHD1*. The gene encodes an epigenetic regulator that physiologically promotes and maintains the heterochromatin status at the *D4Z4* locus [12,28,29]. *DNMT3B* is a de novo DNA methyltransferase and rare variants within this gene have been reported to be associated with FSHD manifestation and penetrance [25]. Recently, *LRIF1* (which codes for a direct interactor of SMCHD1 protein) has been described as a novel disease gene responsible for FSHD [26]. Intriguingly, *LRIF1* biallelic variants have been detected in FSHD-affected subjects. This finding may be in line with a possible autosomal recessive pattern, which is in contrast with the expected autosomal dominant pattern of FSHD. However, such a homozygous variant was detected in a patient born from a consanguineous marriage who also displayed a permissive 4qA haplotype and a *D4Z4* array of 13 RUs. Indeed, this genetic makeup is consistent with other patients carrying pathogenic variants that segregate by an autosomal dominant pattern [25,29,30]. The study does not report any specific data concerning the clinical presentation of parents, except for the mother of the proband, who was reported as healthy [26]. Although the detected *LRIF1* variant has been clearly associated with FSHD, its pattern of inheritance remains elusive because of the lack of sufficient data to determine it. Nevertheless, this issue strongly highlights that even the molecular mechanisms underlying FSHD transmission could be heterogeneous and still need to be fully understood.

Overall, the presence of pathogenic variants in *SMCHD1*, *DNMT3B* and *LRIF1* have been found to be responsible for FSHD in the absence of a contraction of the *D4Z4* array to 1–10 RUs (i.e., DRA). However, pathogenic variants in *SMCHD1* have also been found to act as disease modifiers in the presence of DRA, thus highlighting the existence of a digenic inheritance pattern and of a disease continuum between FSHD1 and FSHD2 [24,27,28,29,31,32]. The above-mentioned information suggests that analyzing the genetic architecture of *D4Z4* together with the mutational landscape of genes involved in the chromatin regulation of this locus could provide a better characterization and a more accurate genotype–phenotype correlation in patients and families with FSHD. For this purpose, the use of NGS approaches represents a powerful tool for sequencing the known genes while simultaneously having the opportunity of identifying variants in novel genes, whose alteration could be responsible for FSHD development and severity.

## 3. Epigenetic Features of FSHD

Epigenetic elements have been investigated for their potential contribution to the pathogenesis of FSHD, as well as to the clinical variability and expressivity of disease. As a matter of fact, the derepression of *DUX4* is allowed by an open chromatin conformation, which is marked by the occurrence of specific epigenetic events, including a local hypomethylated status and histone acetylation markers.

### 3.1. DNA Methylation Status of D4Z4 Array

The DNA methylation status related to the 4q35 locus has been extensively studied and investigated for its contribution to the FSHD expression and severity [12]. On this subject, low methylation levels were found to correlate with the severity of symptoms in 49 symptomatic FSHD individuals carrying *SMCHD1* pathogenic variants [33]. From this point on, the hypomethylation at the *D4Z4* locus in FSHD has been investigated as a potential biomarker able to support the molecular diagnosis [14,34,35,36,37,38,39,40,41]. However, the heterogeneity in terms of methods and cohorts led to controversial results [42,43]. Moreover, different regions across the *D4Z4* array have been studied with the purpose of providing a comprehensive methylation profile that was representative of the entire locus [43]. In particular, the most employed method is represented by bisulfite sequencing (BSS), which allows for the detection of methylated and unmethylated cytosines by sequencing analyses performed after a sodium bisulfite-based treatment on genomic DNA that only converts unmethylated cytosines into uracyles [35,37,39,42,44]. The BSS methods have been used to measure the percentage of methylated CpG sites within each *D4Z4* RU, including the 5′ of *DUX4*-ORF and the distal subtelomeric 4q35 region.

Moreover, techniques based on the methylation-sensitive restriction enzyme (MSRE) take advantage of the presence of methylation-sensitive restriction sites for calculating the methylation levels of the *D4Z4* [33,38,44]. In particular, this technique is based on the analysis of restriction fragments obtained by enzyme digestion and followed by Southern blotting and p13E11-probe hybridization. To this purpose, BsaAI, FseI and CpoI have been used as methylation-sensitive enzymes [42]. In particular, the most utilized MSRE is FseI because its restriction site (localized upstream of the *DUX4*-ORF within each RU of the D4Z4 array) was considered highly informative for the *D4Z4* methylation status [33,38,43].

Another approach for the DNA methylation assessment is based on the utilization of antibodies that specifically bind methylated cytosines. This technique, known as methylated DNA immunoprecipitation (MeDIP), led to the establishment of methyl DNA-antibody complexes that can be purified. As a result, the immunoprecipitated DNA fraction is enriched with the methylated fragments in order to identify differential DNA methylation regions by means of targeted sequence analysis [45]. In particular, this approach has been used to assess the methylation levels related to the 5′ and 3′ of each *D4Z4* RU as well as to a central region harboring the *DUX4* promoter [42].

Despite FSHD patients generally displaying lower DNA methylation levels than control subjects, the correlation of these levels with the magnitude of RU reduction and with the disease severity remains controversial. This aspect may also be due to the different methodologies, cohorts and regions/CpG sites that have been investigated. In fact, certain studies have evaluated the methylation levels of several CpG sites within the RU [33,35], whereas others have highlighted the relevance of the distal sequence, especially of a single CpG located near the PAS, known as CpG6. Indeed, this site was proposed as a discriminating biomarker for FSHD [39]. Moreover, some studies have suggested the use of the D4Z4 methylation status to distinguish FSHD2 patients from FSHD1. In particular, regions at the 5′ of *DUX4*-ORF have shown significantly lower methylation levels in FSHD2 cases [36,37]. Intriguingly, it has been postulated that epigenetic factors regulating chromatin condensation may bind these sequences in order to exert their repressive function [36]. In fact, the known FSHD-associated genes (*SMCHD1*, *LRIF1* and *DNMT3B*) act as chromatin repressors and, thus, directly or indirectly enhance the local DNA methylation status. Therefore, a marked local lower methylation could reflect their loss of function. On this subject, a segment within the 5′ of the *DUX4*-ORF, namely the *DR1* region (containing 29 CpG sites), has been reported to display very low methylation levels in FSHD2 cases [36]. Given these data, the *DR1* region represents a candidate biomarker for providing an in-depth characterization of FSHD patients.

### 3.2. Additional Factors Involved in the Epigenetic Changes at the D4Z4 Array

Among the epigenetic elements able to act on the *D4Z4* array, long non-coding RNAs (lnc-RNA) have been investigated for their potential effect on the *D4Z4* transcriptional status. In particular, DBE-T, whose gene is localized near the *D4Z4* array, can be considered the better-known chromatin-associated lnc-RNA involved in the topological reorganization of the *D4Z4* array. The lnc-RNA DBE-T was detected in FSHD primary muscle cells and biopsies, and it was found to contribute to the local transcriptional derepression by recruiting chromatin activators [46,47].

Moreover, different studies focused on the histone modifications potentially involved in determining chromatin relaxation and aberrant *DUX4*-FL expression [48,49,50]. Notably, Balog et al. 2012 investigated the correlation between the epigenetic status of the *DUX4* promoter with clinical severity and muscle impairment in fibroblasts and myoblasts derived from 15 FSHD patients. In particular, they assessed the ratio between the levels of trimethylation at the lysine 9 of histone 3 (H3K9me3, which is associated with transcriptional repression) and those related to dimethylation at lysine 4 of the same histone (H3K4me2, which is a marker of active chromatin). The authors considered this ratio to be related to the degree of chromatin compaction (chromatin compaction score, ChCS). As a result, this ratio was found to be significantly decreased in patients’ samples (*p* < 0.01) with respect to those derived from five controls, thus highlighting the presence of a more relaxed chromatin at the disease locus in FSHD subjects. Concerning the correlation with the clinical parameters, the ChCS was only found to be negatively associated with the clinical score in fibroblasts, although it failed to reach the statistical significance (*p* = 0.062) and raised the need for further investigation [48].

Indeed, the loss of H3K9me3 at the *D4Z4* array has been widely considered as a mechanism closely involved in the FSHD pathogenesis. Interestingly, a study by Zeng et al. performed chromatin immunoprecipitation (ChIP)-based experiments to reveal that the SUV39H1-dependent H3K9me3 is required for the recruitment of HP1γ/cohesin [49]. In particular, HP1γ plays an important role in transcriptional silencing [51,52]. Zeng et al. noted that SUV39H1-mediated H3K9me3, and the subsequent binding of the HP1γ/cohesin complex, was lost in FSHD. Interestingly, this loss was detected not only at the contracted 4q-*D4Z4* allele but also in the remaining intact *D4Z4* alleles on both chromosomes 4q and 10q. Moreover, the loss of H3K9me3 was detected in different cell types (myoblasts, fibroblasts and lymphoblasts) from FSHD patients only, suggesting that this alteration could represent a general marker of FSHD that can be detected in different biological sources over muscle tissue. Overall, the authors proposed that the loss of H3K9me3 and the related absence of HP1γ/cohesion complex activity at the locus resulted in a detrimental effect on chromatin organization, thereby leading to muscular dystrophy [49]. Intriguingly, the 4q/10q-*D4Z4* specificity of the loss of H3K9me3 was verified in a further study [50]. In fact, this epigenetic alteration was not detected at the other *D4Z4* homologous regions in FSHD myoblasts and fibroblasts. Moreover, they found that the experimental suppression of H3K9me3 was able to impair the binding of SMCHD1 at the *D4Z4* locus, and this was found to enhance the derepression of *D4Z4* with the subsequent increased *DUX4* expression in FSHD-derived myoblasts [50]. Therefore, further research on FSHD primary cells will be useful to better clarify the physiological role of the H3K9me3 on the recruitment of epigenetic regulators at the *D4Z4* array.

Altogether, these data support the relevance of assessing the conformation of *D4Z4* and its three-dimensional changes to better elucidate the mechanisms leading to FSHD development. As a matter of fact, long-range chromatin contacts or high-order spatial genomic interactions have been postulated to change and modulate the expression of the *D4Z4* locus too. Intriguingly, a study on FSHD1 patient-derived myoblasts reported that the presence of DRA could lead to the activation of myogenic factors by changing the spatial organization of these genes within the nucleus [53]. Despite this evidence, the role of such interactions in the pathogenesis and their potential usefulness for the disease characterization deserves to be fully elucidated.

Indeed, the DNA methylation patterns at *D4Z4* could also be influenced by the above-mentioned high-order interactions. For instance, a binding site for CTCF protein is located near the *DR1* sequence. *CTCF* is a known insulator able to shape the three-dimensional conformation of the chromatin in order to limit genomic domains in which genetic and epigenetic elements can tightly interact to regulate the expression of local genes [54]. Of note, the role of *CTCF* in determining the insulation of the *D4Z4* array has been postulated, but the effect of CTCF activity (and dysfunction) on the transcriptional status of *DUX4* remains to be elucidated [55,56].

Overall, it could be very interesting to study if the methylation patterns related to *D4Z4* might reflect alterations of complex spatial genomic interactions and/or the alteration of the function of CTCF and other insulator complexes (such as cohesin) or epigenetic regulators that may potentially contribute to the disease development. On this subject, two studies identified different proteins involved in the epigenetic regulation of the *D4Z4* array [57,58]. By comparing seven human myoblast cell lines with two controls and exploiting an approach that combines ChIP, CRISPR-Cas9 and mass spectrometry (MS), Campbell et al. identified 261 proteins, including known-*D4Z4*-associated factors, cohesin complex components [29,49] and other molecular interactors. Notably, CHD4, HDAC2, MTA2 and RBBP4, which include many of the components of the nucleosome remodeling deacetylase (NuRD) complex, were among the isolated proteins. The authors reported that NuRD and CAF-1 complexes repressed DUX4 expression and that these factors were found to be necessary for maintaining DUX4 transcriptionally inactive in skeletal-muscle-derived cells and induced pluripotent stem cells [57]. Moreover, Goossens et al. investigated novel SMCHD1 interacting proteins in two FSHD cell lines and assessed their functionality in the *D4Z4* repression. This study identified 28 nuclear proteins that potentially interact with SMCHD1 [58]. In particular, the loss of these SMCHD1 interacting proteins, such as RuvB-like 1 (RUVBL1), was found to further derepress *DUX4* in FSHD myocytes. RUVBL1 participates in several protein complexes involved in transcriptional control and chromatin maintenance. Of note, 12 out of 28 proteins (namely, SMCHD1, RUVBL1, HIST1H1C, COIL, HNRNPA1, RAD50, RAD21, HNRNPA0, PRPF8, ALYREF, PRPF19 and MYO1C) are in common with the *D4Z4* chromatin components identified by Campbell et al. (2018) [58].

Such studies investigated the protein interactome occurring at the *D4Z4* array, as it may be useful for identifying novel factors potentially involved in FSHD etiopathogenesis. Furthermore, the identification of multiprotein complexes that regulate DUX4 expression and of additional epigenetic factors linked to FSHD may provide new candidate targets for therapeutic strategies. With this aim, Campbell et al. (2017) focused on signalling pathways and epigenetic machinery that directly or indirectly influence *DUX4* expression in FSHD muscle. They showed that BET (bromodomain and extra-terminal domain proteins, consisting of BRD2, BRD3 and BRD4) inhibitors (BETi) may represent small molecules able to prevent *DUX4* expression in FSHD muscle cells. These data also suggest a possible involvement for the protein BRD4 (and possibly BRD2) in the regulation of the *D4Z4* array [59].

### 3.3. Altered miRNAs in FSHD

Concerning the post-transcriptional regulation, several miRNAs have been studied for their potential alteration in the context of FSHD. On this subject, a study employed murine FSHD models to detect potential miRNA signatures. In this way, an overexpression of miR-31-5p and miR-206 was detected [60]. Instead, a study performed on human FSHD myoblasts observed an overexpression of miR-411 and reported that this miRNA could target a portion of factors involved in the myogenic differentiation [61]. Another study on the same cell type reported 29 miRNAs as dysregulated in FSHD. Notably, the altered expression of miRNAs (such as miR-1, miR-133a, miR-133b and miR-206) involved in muscle homeostasis and differentiation was reported [62]. Furthermore, miRNAs signatures were investigated during an in vitro differentiation process in FSHD primary myoblasts by means of NGS approaches. This study unveiled the dysregulation of 38 miRNAs, a proportion of which were involved in relevant molecular pathways for the muscle homeostasis and function. Interestingly, a lower number of miRNAs were found to be modulated during myogenesis in FSHD compared to control cells, suggesting that an overall dysregulation of miRNAs expression could characterize FSHD [63].

Overall, it is clear that different sources, differentiation stages and methodologies led to different outcomes. Nevertheless, these data support the analysis of non-coding RNAs signatures and of their effect as a powerful source of biomarkers that may be useful for improving the knowledge of FSHD, as well as the research of therapeutic targets. On this subject, a recent study proposed miR-675 as a druggable target to be exploited for counteracting *DUX4* toxic effects. In particular, its induced overexpression appeared to suppress *DUX4* and the expression of its related-targets in FSHD-derived myotubes [64].

## 4. Transcriptome Profiling and Single-Cell Approaches in FSHD

### 4.1. DUX4 Signatures and Transcriptome Analyses

FSHD pathogenesis is strongly linked to the toxic expression of the *DUX4* gene, which is considered to be a fundamental hallmark of disease. However, the detection of its expression in muscle tissue has been challenging due to its variegated and burst-like pattern of expression, which occurs in a small number of muscle cells [65,66]. Of note, it has been estimated that the *DUX4* transcript could be detected in a fraction of cultured myoblasts and myotubes ranging from 1/5000–1/1000 and 1/200, respectively [67,68,69]. Despite this low concentration, the aberrant expression of DUX4 in skeletal muscle has been found to lead to the dysregulation of tissue homeostasis [7,70]. On this subject, induced high levels of DUX4 in both immortalized and primary myoblasts, as well as in animal models, were found to be responsible for the activation of transcription of several target genes mainly involved in RNA metabolism and apoptosis [7,70]. Given the low levels of DUX4 in human FSHD muscle, these data and the related mechanisms had to be verified. In this regard, a study developed a murine model able to recapitulate the peculiar DUX4 pattern of expression. As a result, low levels of DUX4 were shown to induce the damage and necrosis of muscle fibers, the infiltration of inflammatory cells and the increased deposition of the extracellular matrix, which have also been reported for FSHD muscles [71]. Considering this information, the dissection of these pathogenetic mechanisms at the molecular level may be relevant for clinical and therapeutical purposes.

Indeed, many research efforts have been focused on identifying DUX4 target genes by inducing its overexpression. On this subject, different studies on myoblasts showed hundreds of genes dysregulated by DUX4 [67,72,73,74,75]. Overall, several of these genes were found to be involved in the early programming of the embryo cleavage stage, immunity, inflammation and regulation of retroelements [67,72,73,74,75]. Therefore, the aberrant expression of *DUX4* has been proposed to trigger toxicity by reactivating the early embryonic program in the adult differentiated muscle tissue [75,76,77]. On this subject, it has been found that *DUX4* shares the ability of activating germ line genes with the mouse ortholog *Dux*. This physiological function is maintained across these species, although DUX4 and *Dux* were found to display different DNA binding motifs within the homeodomains. Indeed, this divergence was found to lead to the transcription of different retroelements [72]. Importantly, the conserved embryonic functions highlight the relevance of DUX4 in the early development. This observation, together with the results of the above-mentioned studies [67,72,73,74], further supports the investigation of the effects of DUX4 on the reactivation programming in adult muscles. Besides the data concerning the function of identified target genes, these studies showed a high discrepancy. In fact, Banerji and Zammit, 2021 estimated that only eight targets, namely *ZSCAN4*, *TRIM43*, *RFPL1*, *RFPL2*, *RFPL4B*, *PRAMEF1*, *PRAMEF2* and *PRAMEF12*, have been commonly detected by these studies. Moreover, the silencing of genes targeted by PAX7 (due to *DUX4* activation) has been proposed as a pathological hallmark of muscle degeneration in FSHD [68,78].

The identification of FSHD-related gene expression profiles may be important for clinical purposes. In fact, genes targeted by DUX4 may represent signatures able to differentiate FSHD-affected patients from other subjects, as well as potential markers of disease activity and progression. Moreover, these signatures may represent therapeutic targets to be exploited for counteracting *DUX4* toxic effects and for evaluating the response to drugs. Therefore, research studies have been conducted on muscle biopsies from FSHD patients to validate the dysregulation of DUX4-induced factors and, more generally, to identify gene expression patterns related to FSHD. However, the analysis of a bulk tissue has often led to the detection of spurious profiles reflecting the average of heterogeneous cellular populations. In fact, only a proportion of cells are able to express the stable form of *DUX4*-mRNA (namely, the *DUX4*-FL) in FSHD-affected muscles. This pattern of expression leads to the establishment of a mixture of DUX4-positive and negative cells with distinct epigenomic, genetic and transcriptomic features. Interestingly, all of these cells can also display other FSHD-associated markers (such as the *PAX7* signature), independently from the positivity to the DUX4 expression [79,80]. Supporting this data, the presence of FSHD-related gene expression profiles was assessed in patients’ muscle tissue by means of microarray-based technologies, reporting a globally low differential gene expression (fold change < 1.5) [81]. Similarly, Yao et al. (2014) performed RNA-seq on DUX4-overexpressing myoblasts and myotubes [73], as well as on muscle biopsies from 15 FSHD individuals and 9 controls. In particular, they detected 90 and 348 potentially upregulated target genes in myoblasts and myotube cells, respectively. However, the authors reported that the DUX4 target genes were not found among the differentially expressed genes (DEGs) in six FSHD biopsies. Moreover, they highlighted that moderately expressed targets may have not been detected as DEGs due to the presence of cells not prone to *DUX4* expression. In addition, the difficulty in detecting such targets in the myoblast and myotube cells could also be due to a contamination of a control sample with a DUX4-induced RNA sample, as illustrated in the study by Young et al. (2013) [72,73].

Interestingly, gene expression profiles were investigated to assess their utility for assessing the disease activity and the prognosis. In particular, a study performed on biopsies from 36 FSHD patients found that DUX4 signatures were significantly upregulated in muscles positive to short tau inversion recovery (STIR+, which is a marker of muscle pathology) compared to normal muscles subjected to MRI assessment (*p* < 0.001). Interestingly, the authors reported that 10 biopsies (characterized by histological marks of mild/moderate pathology) did not show DUX4 signature dysregulation, although they displayed altered factors involved in immunity and extracellular matrix organization, which are actually known as DUX4 targets. Given these data, the DUX4 signatures may not be easily detectable in the early phase of FSHD activity [82].

The usefulness of FSHD-associated transcriptome signatures as biomarkers of disease progression has been evaluated monitoring the muscles of the previously mentioned 36 patients over 1 year [83,84]. In particular, Wong et al. did not find significant differences in muscle pathology and gene expression profiles over 1 year. In addition, this work further validated the relation between DUX4 signatures and advanced disease activity. In fact, it reported 164 differentially expressed genes in 17 mild FSHD-affected muscles compared to 8 controls (*p* < 0.05). Of them, 52 genes were able to effectively discriminate mildly affected muscles (AUC-ROC: 0.9) [83]. Banerji., 2020 exploited 26 of the 36 above-mentioned human samples to evaluate the PAX7 signature, which was previously found [78] as a prognostic marker for FSHD. In this study, a significant difference (*p* = 0.038) was found concerning the levels of PAX7-repressed genes between 2-year and 1-year muscles. Interestingly, this study proposed the PAX7 signature as a marker of short-term progression, given its ability to reflect low-level alterations and subtle molecular changes associated with disease activity [84].

Overall, these studies found that transcriptome signatures may reflect alterations of muscle homeostasis and pointed out the importance of finely characterizing gene expression alterations at early times of disease activity in order to draw a trajectory of disease. On this subject, it may be useful to investigate if higher resolution approaches, such as single-cell transcriptome analyses, may provide a more sensitive detection of the subtle molecular changes, as well as facilitate the identification of the specific cell types displaying such alterations.

### 4.2. Single-Cell and Single-Nucleus Transcriptome Analyses

Based on the illustrated data, it is clear that the complex molecular background, as well as the peculiar genetic defects and epigenetic changes underlying FSHD, raised the need for better characterizing the global transcriptional landscape at a single-cell-level [85,86].

Overall, several efforts have been made concerning the development of technologies that provide large-scale molecular characterization at a single-cell resolution [87,88,89,90]. This approach could help to tackle the cellular heterogeneity of the pathological tissue, in terms of differences at the level of DNA, RNA, proteins and metabolites [91,92,93,94,95].

Single-cell RNA-sequencing (scRNA-seq) combines the investigation of the whole transcriptional profile with comprehensive bioinformatic and computational approaches to explore the molecular signatures and gene regulatory networks for specific cell types in the context of a tissue [96]. Firstly, the scRNAseq workflow requires the isolation of single cells that can be performed by means of manual fluorescence-activated cell sorting (FACS) or by using microfluidics-based systems. The next steps of RNA extraction, cDNA amplification, library preparation and sequencing are in common with the bulk RNA-seq methods. Secondly, the analysis of data obtained from scRNA-seq is a crucial step and needs appropriate computational and statistical methods to ensure a reliable and proper interpretation [97].

The application of the scRNA-seq approach could provide novel and more accurate insights into the disease pathogenesis, as well as new perspectives for understanding the genotype–phenotype correlations, allowing for the identification of specific FSHD cellular subtypes affected by certain genetic signatures [96]. On this subject, different studies showed the utility of scRNA-seq in studying FSHD etiopathogenesis and provided interesting results [79,80,86,98].

Starting from tissue-cultured human primary myocytes from four FSHD patients (two FSHD1 and two FSHD2) and two healthy controls, van den Heuvel et al. (2019) reported differences among the FSHD and control tissues and profiled transcriptome signatures in the *DUX4* expressing and non-expressing FSHD cells, revealing more than 1300 DEGs [79]. Interestingly, only 231 were in common with the genes previously identified by Yao et al., 2014 [73] and Rickard et al., 2015 [66]. This discrepancy may be due to the above-mentioned difficulties in detecting DUX4-target genes or the major resolution given by the single-cell analysis. Moreover, authors selected a restricted set of 49 genes to generate a “pseudotime” trajectory model with the aim of evaluating the progression of FSHD at transcriptome level. This analysis is based on specific algorithms able to process data collected at multiple time points with the aim of studying the alteration of physiological processes (such as cell differentiation and proliferation) and the pathogenetic changes over time at a single-cell resolution level [99,100].

Furthermore, another study by Jiang et al., 2020 performed a single-nucleus RNA-seq on muscle from a FSHD2 patient and a healthy control in order to study the DUX4-expression effects in the muscular syncytia, highlighting how affected nuclei are quite different from each other in terms of transcriptional profiles. In particular, this study highlighted that a very exiguous number of nuclei within the same myotube was characterized by the expression of *DUX4* [86].

Intriguingly, Banerji and Zammit, 2019 focused the attention on the *PAX7* signature and reported it as a powerful classifier for FSHD samples based on the sc-RNAseq data published by Van den Heuvel et al., 2019 [79,80].

Recently, a study aimed at evaluating human induced pluripotent stem cells (hIPSCs)-derived myoblasts as disease-representative models for neuromuscular conditions obtained such cells from three FSHD1 individuals. By performing sc-RNAseq during the differentiation process from myoblast to myotubes on both FSHD and control-derived cells, a proportion of DUX4 targets (*MBD3L2*, *TRIM43*, *LEUTX* and *ZSCAN4*) were detected as overexpressed in FSHD subjects [98].

Sc-RNA-seq and Sn-RNA-seq studies may overall lead to the identification of disease-associated gene expression profiles that could also be exploited for clinical purposes, enabling the identification of candidate biomarkers for diagnosis and/or disease staging, as well as druggable targets. Nevertheless, considering that different pipelines for scRNA-seq are available nowadays, a standardization of the available analytical methods should be recommended [101].

Moreover, despite the high informativeness and specificity of these analyses performed on muscular tissue, the invasiveness of clinical biopsy raises the need for employing an easy-to-access biological source that could facilitate the clinical application. Indeed, a FSHD-specific lymphoblast signature of 237 up-regulated genes (of which, 10 were DUX4 target genes) was identified on immortalized B-lymphoblastoid cell lines obtained from the whole blood of three FSHD patients and three family controls. In particular, this signature was confirmed in muscle biopsies and was found to be associated with DUX4 activation and the early tissue infiltration of immune cells. These data support the occurrence of the FSHD transcriptome signature in blood and intriguingly shed light on a potential driver role of inflammatory/immune cells in the etiopathogenesis on FSHD [102].

In addition, a study that performed RNAseq on whole blood samples from 54 FSHD patients and 29 healthy controls observed the absence of significant DEGs, including *DUX4*- and *PAX7*-related signatures, between the two groups. Indeed, authors reported that a proportion of 34 genes with a nominal association failed the multiple correction tests [103]. Altogether, these data highlight the need for performing finer analyses to evaluate the presence of specific FSHD transcriptome signatures in blood. Thus, research efforts addressed at finely defining DUX4-mediated cascades and identifying FSHD-associated transcriptome signatures may benefit from single-cell approaches applied to an accessible biological source.

## 5. Machine-Learning Application to Support the Disease Characterization and Diagnosis

### 5.1. Artificial Intelligence (AI) and Machine Learning (ML) in Medicine

AI is an umbrella term that includes technologies that can solve tasks requiring human intelligence. ML is one of the main branches of AI and it includes algorithms that autonomously learn from data to make decisions. In particular, ML algorithms for diagnosis can be trained using a supervised learning approach to associate a label (e.g., affected vs. non-affected) with the corresponding input data. The establishment of AI-based methods in clinical diagnostic protocol aims at providing more precise diagnoses with a fast, unbiased and data-driven evaluation of patients [104]. To date, AI has enhanced clinical diagnosis and decision-making performance in several medical domains, such as oncology, cardiology and neurology [104,105]. ML-based methods have been tested for enhancing the usefulness of molecular disease biomarkers, including genetic and epigenetic signatures in different kinds of diseases and phenotypes [106,107,108,109,110]. In fact, ML algorithms can be set to build computer-aided diagnosis (CAD) tools, user-friendly software that aids physicians with AI predictions. CAD systems that exploit data from multiple sources are more accurate when highly complex models are implemented, such as deep neural networks. These are AI algorithms that mimic brain functioning. Therefore, they are made of multiple layers of nodes that apply complex transformation functions to the input data. Due to this complexity, their behavior is uninterpretable, but CAD tools can be enriched with explainable AI (XAI) methods [111], allowing humans to better understand how the algorithm made its predictions, ultimately fostering physicians’ trust in AI and its spread in clinical setting [112].

### 5.2. Existing Artificial Intelligence Applications to FSHD

To date, ML approaches to FSHD data analysis have mainly focused on magnetic resonance imaging (MRI) or gene expression data. In the MRI-based AI modeling, upper/lower limb muscles MRI images are usually extracted and quantitative measures such as fat fraction (FF) and water T2 (wT2) are computed, but expert radiologists have been able to include up to 47 radiological features in their dataset. These measures are then used as an input for one or more ML algorithms to obtain common precision medicine deliverables, such as biomarkers identification, diagnosis or prognosis. To date, only one study using a support vector machine (SVM) obtained a 0.89 accuracy with 95% (CI 0.85–0.92) in discriminating FSHD cases from patients affected by other myopathies [113]. MRI-based algorithms have also been used to predict functional outcomes in FSHD, such as the wheelchair use. In this context, a random forest trained on clinical and genetic longitudinal data achieved a 0.79 accuracy and 0.85 AUC [114]. Despite there being few studies, the MRI-based AI modeling of upper/lower limb muscles provided promising results in FSHD and confirmed the diagnostic/prognostic role of FF and wT2 upper/lower limbs features. These two features are commonly derived from qualitative imaging, requiring the manual interpretation of the weightening and contrasts of the images. Quantitative MRI (qMRI) could be useful for standardizing measurements of FF and wT2 upper/lower limbs features, but the required MRI sequences are not routinely available in every FSHD center. ML was used to overcome this issue and a random forest was used to predict the qMRI values of FF and wT2 from conventional MRI, obtaining mean absolute errors ranging from 0.110 to 0.133 for FF and 0.068 to 0.115 for wT2 [115]. The few applications that have been found, coupled with their highly variable cohort sizes (ranging from 14 to 558), suggest that FSHD research would benefit from consortium initiatives similar to the Parkinson’s Progression Markers Initiative or the Alzheimer’s Disease Neuroimaging Initiative, to finally enter into the big data era. A common effort in the harmonization of data sharing and standardization practices would be beneficial for the advent of AI and CADs in FSHD based on reliable high-quality data [116].

Contrary to MRI-based AI models, which have mostly been used for differential diagnosis or prognosis, AI models based on transcriptomics are used to explore the potential of molecular diagnosis of FSHD. In these tasks, FSHD and healthy controls (HC) are compared to identify differentially expressed genes (DEGs). Subsequently, the DEGs will be used as an input in an ML algorithm that will classify observations in FSHD or HC. Generally, this kind of study relies on RNA extracted from muscular biopsies, which are relatively more difficult to obtain than other more easily available biological sources, such as blood samples. Nevertheless, AI models taking DEGs from muscle tissue as an input give a high accuracy for both biceps (0.90) and deltoids (0.80) using L_1_-regularized logistic regression [81]. A similar level of accuracy (0.91, 95% CI [0.907–0.913]) was yielded using an SVM to diagnose FSHD on gene expression data from skeletal muscle biopsies, whereas a previous SVM application on the same dataset reported an impressive 0.994 accuracy [117,118]. Even though there is only one published experiment where FSHD is diagnosed using gene expression data from blood samples, the results are promising, with a logistic regression achieving a mean AUC ranging from 0.794 (95% CI [0.618–0.961]) to 0.883 (95% CI [0.735–1.0]) [103].

**Figure 2:**
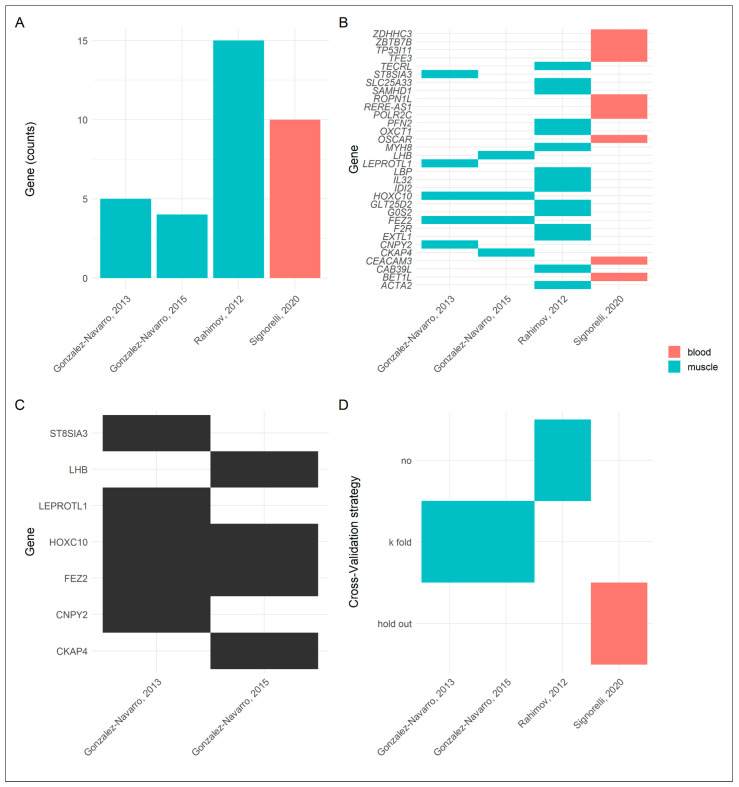
A comparison of gene-expression-based AI models with data attained from (Gonza-lez-Navarro et al., 2013 [117], Gonza-lez-Navarro et al., 2015 [118], Rahimov 2012 [81] and Signorelli 2020 [103]). (**A**) Bar plot indicating the number of DEGs found in the considered studies. Up to 15 DEGs representing potential biomarkers for FSHD were identified across 4 studies. (**B**) Tile plot indicating which genes were found by the considered studies. All of the genes identified in blood (red) were not retrieved in the other studies based on muscle biopsies (blue), and this may be due to the different levels of gene expression of the two biological samples. However, only two genes of 15 were identified both from Gonzalez-Navarro et al., 2013 and Gonzalez-Navarro et al., 2015 across the experiments performed on muscle data. (**C**) Tile plot with a focus on the studies by Gonzalez-Navarro et al. The studies proposed by these authors were performed on the same dataset and used the same SVM algorithm. Despite these favorable conditions, only 2 out of 7 DEGs were confirmed. (**D**) Tile plot to visualize the cross-validation strategies used in FSHD vs. HD modeling, suggesting that the performance metrics used may be inflated by the low sample size (12 < *n* < 54).

Up to 15 DEGs have been found in these experiments (Figure 2A), and they could all be considered as biomarkers of FSHD. Most of the DEGs identified in blood are non-overlapping with those found in muscle gene expression data (Figure 2B). This finding may advocate for a multi-source data integration when it comes to using gene expression data in an ML pipeline. Unfortunately, these DEGs should not be considered a stable molecular signature of the disease, since most of them were not confirmed by other studies adopting the same data source and experimental design (Figure 2B). Furthermore, only two out of seven DEGs (*FEZ2* and *HOXC10*) were confirmed by the same group using the same dataset (E-GEOD-3307) and the same SVM algorithm (Figure 2B,C). Taking into consideration the instability of these candidate biomarkers, the performance of the models trained with these DEGs should be reconsidered (accuracies ranging from 0.790 to 0.994). A possible explanation is that ML requires appropriate cross-validation strategies when the sample size is low, and the k-fold or hold-out validation strategies used in these studies inflated the performance metrics (Figure 2D). As a matter of fact, only one study included more than 15 subjects. This considered, these analyses are lacking stability and do not seem reliable in detecting DEGs as effective biomarkers.

### 5.3. Multi-Source Data Integration in AI for Medicine and FSHD Research

As presented above, AI applications for FSHD research focused on data from a single source. To the best of our knowledge, there are still no published attempts to train an AI model with multi-modal data in FSHD research to date. Research trends in other pathologies, such as cancer and neurodegenerative diseases, have shown that multi-source data integration follows years of published applications on single-source data, as those are easier to implement [119,120]. Nevertheless, the complexity of such a disease deserves a multifaceted view of the patients’ biological and clinical states. In fact, it has been extensively shown that integrating multi-source data when developing AI for medicine gives more accurate models. This multi-source data integration paradigm opens a greater understanding of disease-specific mechanisms and more reliable predictive models to be used in CAD systems. Most AI for medicine applications exploiting integrated multi-source data implement deep neural networks due to their ability to manage highly non-linear associations between the input data and the predicted outcome. As an example from Alzheimer’s Disease research, it has been shown that the integration of MRI with PET imaging, cerebrospinal fluid and genetic variants achieves up to 10% higher accuracy levels. To perform an efficient multimodality fusion, a system of extreme learning machine models (basically neural networks) was applied, combining the information from all different sources and finally providing its prediction [121]. In a similar fashion, cancer subtype classification can be performed with a system of deep neural networks to integrate multi-omics data. It has been shown that multi-omics data integration improves the model’s performance compared to using single-omic data [122]. This considered, we believe that FSHD research would benefit from multi-source data integration, as appropriate AI algorithms can manage their complexity and detect relevant mechanisms invisible to the human expert, finally giving clinically useful insights. When multi-source data integration is trending in FSHD research too, deep neural networks will also take the field, unleashing their unparalleled power in data elaboration to manage healthcare big data integration complexity at best. To make this dream come true and achieve the best results in FSHD research, there is a need for the availability of clinical data from medical health records, multi-omics molecular data from muscle tissue biopsies and blood and medical imaging data from cellular microscopy and MRI. All of this can be integrated and also followed along in time, leading to the ability to monitor FSHD over its evolution, opening the blinds on disease characterization, prediction of its progression and selection of treatments.

To date, the utilization of molecular data and ML approaches has not been deeply evaluated in large cohorts. In particular, there are still no published attempts of FSHD classification based on DNA methylation data. Our group is currently working on a highly curated ML analysis of FSHD classification on methylation data related to the *D4Z4* array, with promising results for improving the disease characterization (manuscript in preparation). In fact, the exploitation of such data may help to tackle the challenges in FSHD identification and its differential diagnosis with other neuromuscular diseases that may be characterized by overlapping phenotype features, such as limb girdle muscular dystrophy (LGMD). This can be useful for those patients that are harder to diagnose due to a subtler disease pathophysiology, lowering both false positive and false negative error rates. Moreover, this could result in lower costs for clinical centers, reducing inappropriate accesses to specialist visits thanks to an accurate and reliable omics screening phase carried out with AI.

Indeed, the integration of such data with those from genomics and transcriptomics, along with clinical records and demographics, has yet to be applied in the development of AI-based CAD tools for FSHD [120,123].

**Figure 3:**
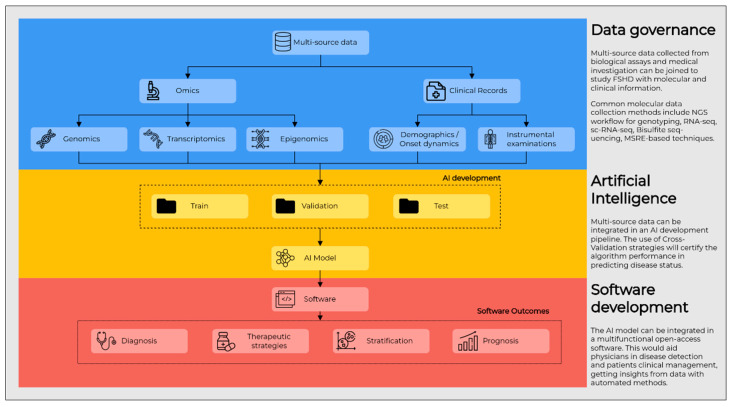
Schematic overview of a multifunctional tool. Such a tool would be able to integrate molecular, clinical and phenotype data with AI pipelines for enhancing the knowledge of FSHD and foster the research for novel treatment strategies, as well as biomarkers to be applied for the characterization, diagnosis, prognosis and monitoring of disease.

Combining and analyzing multimodal data to train accurate models and XAI methods to investigate their behavior would finally lead to highlighting relevant mechanisms underlying FSHD pathogenesis. The development of software tools would be advantageous for the specialists involved in FSHD patients management. The ideal AI-based tool would be able to take into account data from different sources, such as genetic variants, *D4Z4* size and haplotype, DNA methylation status and FSHD-related transcriptome signatures, MRI, clinical records and demographics, finally providing predictions and summaries to clearly show the patient status. This would facilitate a proper monitoring of the disease stage over time in the FSHD trajectory. Thus, not only would such a software aid the diagnosis, but it would also give support in patients’ stratification and prediction of prognosis, as well as in the choice of therapeutic strategies, thus constituting a proper multifunctional tool. Of note, considering that unknown targets and mechanisms could be discovered concerning FSHD pathogenesis, such a tool should be flexible and open to implementations with novel data (Figure 3).

## 6. Discussion

The present review discussed the molecular features and technologies able to produce genetic and epigenetic data, which could be combined with detailed clinical information into an advanced multifunctional tool built by ML and AI approaches. Indeed, the availability of functional tools and molecular tests able to standardize and optimize the diagnosis, prognosis and treatment of FSHD are crucial for coping with the existing phenotypic and genetic variability among patients and families. The molecular signatures and analytical methods discussed above have been summarized in Table 1.

Currently, the disease heterogeneity and variable expressivity is likely to be characterized by a complex molecular scenario that has been partially disclosed (Figure 1). Currently, the genetic diagnosis is based on the assessment of DRA and/or pathogenic variants within FSHD-associated genes, although these approaches do not always provide a complete and exhaustive diagnosis. As a matter of fact, the pathogenic size ranging from four to eight RUs has been reported in 3% of the healthy general population [124]. Moreover, the potential occurrence of FSHD-associated pathogenic variants in patients carrying a borderline DRA (8–10 RU) supports the clinical utility of performing additional genetic analysis in such cases. In fact, this approach could be very helpful in assessing possible “multigenic” inheritance patterns (namely, the co-occurrence of DRA, 4qA haplotype, pathogenic variants within different genes and epigenetic alterations) responsible for the variable expressivity and severity of disease in some patients or within families [28,125]. In fact, this RU range actually represents a “gray zone” for which the genetic diagnosis should be carefully conducted taking into account the presence of variable phenotypes contributed by the simultaneous presence of DRA, epigenetic and other genetic alterations. As a matter of fact, a possible relevance of epigenetic mechanisms was highlighted in the past by observing that FSHD-affected monozygotic twins displayed different degrees of severity although sharing the same FSHD-associated genetic features [126]. As discussed in the present manuscript, several research studies were able to assess the key role of epigenetic elements in establishing and modulating FSHD phenotypes. Therefore, their implementation into the clinical practice could be helpful for improving the characterization of patients and supporting the molecular diagnosis. As above-mentioned, different studies highlighted that the DNA methylation status of the *D4Z4* locus could be helpful in distinguishing FSHD-affected subjects, but also FSHD subjects carrying variants from the others [34,35,36,37]. However, considering the lack of agreement concerning the diagnostic power of *D4Z4* methylation, further studies are still necessary to evaluate it as a useful biomarker and consider its application in the perspective of creating a multifunctional tool for FSHD characterization. To date, this objective can be more pursued thanks to technological progress. In fact, methylation analysis could be performed on samples retrieved from different sources by means of different technologies able to provide the required resolution (single-base or whole region) and throughput (targeted or genome wide). For instance, affinity enrichment-based methods, such as MeDIP, allow for a large-scale evaluation of methylation patterns, whereas the BSS-based methods could be employed when a higher resolution, at a specific target, is required [127].

Bearing in mind the complex etiopathogenesis underlying FSHD, of which, a fundamental hallmark is the expression of *DUX4*, the assessment of transcriptome signatures strictly related to FSHD and particularly to the effects of *DUX4* activation is of paramount importance for the characterization of disease and the research of clinically useful diagnostic, prognostic and therapeutic markers. In fact, the investigation on transcriptome signatures at a single-cell level could lead to a finer knowledge on FSHD-related patterns of gene expression. This approach could enable the identification of biomarkers to be exploited for the classification of FSHD patients, the staging of disease progression or the design of therapeutic approaches aimed at counteracting the disease (such as DUX4-targeting drugs). Of note, transcriptome data could be exploited with the purpose of translating the dysregulation of gene expression from the cellular level to the tissue level and could thus provide a picture of the muscles compromised by the disease. This condition can be visualized by means of MRI, which allows for the deep phenotyping of FSHD patients [82,128].

Furthermore, given the cellular heterogeneity characterizing the skeletal muscle tissue, it would be useful to utilize sc-RNAseq approaches and, in particular, sn-RNA-seq approaches, which allows for the analysis of multinucleated fiber and, thereby, a higher detection of the disease-related transcriptome signature and molecular disease mechanisms [129]. Overall, the advent of NGS analyses allowed the generation of a huge amount of data, such as the above-mentioned RNAseq data, that could be exploited for clinical purposes. Their analysis requires advanced computational methods that can help the professionals in their interpretation and integration. Thus, considering this and the described difficulties related to the interpretation of genetic analyses, it would be interesting to evaluate the application of AI-based tools for the analysis and integration of the different molecular signatures associated with FSHD. In fact, the utilization of such approaches could improve the selection of useful biomarkers, allowing for a better comprehension of disease features, ultimately enabling a better characterization of affected patients. Furthermore, data obtained from genomic, epigenetic and fine transcriptome analysis of FSHD patients could be combined with such methods for building highly performant classification models, which may be able to discriminate between affected and non-affected subjects, as well as distinguish patients suffering from other neuromuscular conditions.

For this purpose, the integration of clinical and other instrumental data with the information provided by these classifiers will be fundamental in providing accurate genotype–phenotype correlations, supporting physicians in the diagnosis, prognosis and selection of the possible therapeutic treatments or providing access to clinical trials (Figure 2). Indeed, the combination of AI-based tools with FSHD-specific molecular profiles will pave the way for building multifunctional tools able to analyze and integrate FSHD-related molecular and phenotype data tailored to increasing the knowledge of disease pathophysiology and progression and, subsequently, developing novel effective treatment strategies.

In conclusion, the present review highlights how FSHD1 and FSHD2 should not be considered as distinct forms, and rather as part of a disease continuum characterized by a molecular spectrum of genetic and epigenetic factors, whose alteration plays a differential role on *DUX4* repression and, subsequently, contributes to determining the FSHD phenotype. In this scenario, the application of NGS-based technologies is expected to set the basis for providing patients and families with accurate genotype–phenotype correlations and, in parallel, dissecting the different facets of FSHD.

## Figures and Tables

**Table 1:** Overview of the described investigated FSHD molecular signatures and corresponding analytical methods.

Molecular Analysis	Molecular Signature	Methodology	References
***D4Z4* sizing**	DRA, 4q subtelomeric alleles and haplotypes	Southern blot + PFGE+ probes hybridization	Lemmers et al., 2007 [6], Lemmers et al., 2017 [18]
	DRA, 4q subtelomeric alleles and haplotypes, complex rearrangements	MC	Nguyen et al., 2019 [19], Nguyen et al., 2017 [21], Vasale et al., 2015 [22]
		SMOM	Dai et al., 2020 [24]
**Detection of pathogenic variants within FSHD-associated genes**	*SMCHD1*	WES	Mitsuhashi et al., 2016 [27], Lemmers et al., 2012 [29]
	*LRIF1*	Direct resequencing + WES	Hamanaka et al., 2020 [26]
	*DNMT3B*	WES	van den Boogaard et al., 2016 [25]
**DNA methylation**	5′ *DUX4*-ORF	BSS	Jones et al., 2015 [35], Gould et al., 2021 [37], Calandra et al., 2017 [39], Gaillard et al., 2014 [42], Roche et al., 2019 [44]
		MSRE	Lemmers et al., 2015 [33], Nikolic et al., 2020 [38]
		MeDIP	Gaillard et al., 2014 [42]
	*D4Z4* RU	BSS	Jones et al., 2015 [35], Gould et al., 2021 [37], Calandra et al., 2017 [39], Gaillard et al., 2014 [42], Roche et al., 2019 [44]
	*DUX4* promoter	MeDIP	Gaillard et al., 2014 [42]
	Distal *D4Z4* region	BSS	Jones et al., 2015 [35], Gould et al., 2021 [37], Calandra et al., 2017 [39], Gaillard et al., 2014 [42], Roche et al., 2019 [44]
		MeDIP	Gaillard et al., 2014 [42]
**Non-coding RNAs**	lncRNA DBE-T	qRT-PCR	Cabianca et al., 2021 [47]
	Differentially expressed miRNAs	qRT-PCR	Nunes et al., 2021 [60], Harafuji et al., 2013 [61], Dmitriev et al., 2013 [62]
		Small RNA seq	Colangelo et al., 2014 [63]
**Histone modifications**	H3K9me3:H3K4me2 ratio	ChIP	Balog et al., 2012 [48]
	H3K9me3	ChIP	Zeng et al., 2009 [49], Zeng et al., 2014 [50]
**Epigenetic regulators of the *D4Z4* locus**	*D4Z4*-associated proteins	enChIP + MS	Campbell et al., 2018 [57]
	Novel SMCHD1 interacting proteins	SILAC + MS	Goossens et al., 2021 [58]
**Spatial genome organization**	*D4Z4* 3D organization and spatial contacts	4C-seq	Cortesi et al., 2019 [53]
**Transcriptome**	*DUX4* mRNA	qRT-PCR	Dixit et al., 2007 [65], Snider et al., 2010 [69]
	DUX4 target genes	Microarray	Geng et al., 2012 [67]
		RNA-seq	Young et al., 2013 [72], Yao et al., 2014 [73], Choi et al., 2016 [74], Banerji et al., 2017 [78], Signorelli et al., 2020 [103], Wang et al., 2019 [82] Wong et al., 2020 [83]
		ScRNA-seq	van den Heuvel et al., 2019 [79], Guo et al., 2022 [98]
		SnRNA-seq	Jiang et al., 2020 [86]
	PAX7 target genes	RNA-seq	Banerji et al., 2017 [78], Signorelli et al., 2020 [103], Banerji et al., 2020 [84]
		ScRNA-seq	Banerji et al., 2019 [80]

DRA: D4Z4–reduced allele; WES: whole exome sequencing; PFGE: pulse-field gel electrophoresis; MC: molecular combing; SMOM: single molecule optical mapping; BSS: bisulfite sequencing; MSRE: methylation-sensitive restriction enzyme-based technique; MeDIP: methylated DNA immunoprecipitation; qRT-PCR: quantitative real time reverse transcription-polymerase chain reaction; RNA-seq: RNA-sequencing; 4C-seq: chromosome conformation capture (3C)-on-chip; ChIP: chromatin immunoprecipitation; enChip: CRISPR/Cas9 engineered chromatin immunoprecipitation; MS: mass spectrometry; SILAC-MS: stable isotope labelling of amino acids in cell culture mass spectrometry; ScRNA-seq: single cell RNA-seq; SnRNA-seq: single nucleus RNA-seq.

## Data Availability

The presented data are included in the manuscript.
